# Ultrasonography and clinical outcomes following anti-incontinence procedures (Monarc vs MiniArc): A 3-year post-operative review

**DOI:** 10.1371/journal.pone.0207375

**Published:** 2018-12-04

**Authors:** Tsia-Shu Lo, Sandy Chua, Yiap Loong Tan, Ma. Clarissa Patrimonio, Leng Boi Pue

**Affiliations:** 1 Department of Obstetrics and Gynecology, Chang Gung Memorial Hospital, Keelung, Medical Center, Keelung, Taiwan, Republic of China; 2 Division of Urogynecology, Department of Obstetrics and Gynecology, Linko, Chang Gung Memorial Hospital, Linkou Medical Center, Taoyuan, Taiwan, Republic of China; 3 Chang Gung University, School of Medicine, Taoyuan, Taiwan, Republic of China; 4 Fellow, Division of Urogynecology, Department of Obstetrics and Gynaecology, Chang Gung Memorial Hospital, Linkou Medical Center, Taoyuan, Taiwan, Republic of China; 5 Department of Obstetrics and Gynecology, Cebu Institute of Medicine- Cebu Velez General Hospital, Cebu City, Philippines; 6 Department of Obstetrics & Gynecology, Kuching Specialist Hospital, KPJ Healthcare, Kuching, Sarawak, Malaysia; 7 Department of Obstetrics and Gynecology, Dr. Pablo O. Torre Memorial Hospital, Bacolod City, Philippine; 8 Department of Obstetrics & Gynecology, Subang Jaya Medical Center, Ramsay Sime Darby Health Care, Selangor, Malaysia; University Medical Center Utrecht, NETHERLANDS

## Abstract

**Objective:**

To compare the ultrasonographic positional changes of mid-urethral sling(MUS) tape in relation to symphysis pubis, and the different clinical outcomes among women who underwent MUS insertion with MiniArc^TM^ or Monarc^TM^ for the treatment of stress urinary incontinence 3 years after.

**Materials and methods:**

A retrospective follow-up study on patients with clinically confirmed stress urodynamic incontinence and urodynamic stress incontinence who had undergone MiniArc or Monarc surgery. Data regarding preoperative evaluation, intraoperative complications and post-operative follow-ups were collated. Main outcome is to determine the change in position of the sling through measurement of the x- and y-axis at rest and during Valsalva maneuver using the 3D introital ultrasound.

**Results:**

A total of 138 patients were evaluated, 82 belonged to Monarc and 56 to MiniArc. At 3years, objective and subjective cure rates for MiniArc and Monarc were comparable (88%, 91%; p>0.05; 83%, 89%, p>0.05 respectively). Ultrasonographic changes between MiniArc and Monarc from 6 months to 3 years, showed MiniArc to exhibit significant movement in both x- [3.0 ±0.4 mm vs. 2.2 ±0.3 mm (p = 0.02) at rest; 2.6 ±0.3 mm vs. 1.6 ±0.3 mm (p<0.001) during valsalva] and y-axis [3.5 ±0.5 mm vs. 2.0 ±0.3 mm (p<0.001) at rest; 3.3 ±0.5 mm vs. 2.9 ±0.3 mm (p = 0.037) during Valsalva]. The mobility of MiniArc was significantly more than Monarc from rest to Valsalva (1.1 ±0.4 mm vs. 0.3 ±0.3 mm, p = 0.001). Tightness of the sling assessed from the major and minor axis of the urethral core had no significant difference in both groups at rest and during Valsalva. Urethral kinking percentage and the location of the sling did not yield statistical difference.

**Conclusion:**

Maintenance of continence rates of mid-urethral slings depends on the compressive effect of the sling on the urethra, urethral kinking, and sling fixation. From 6months to 3 years, MiniArc changed its position in both x- and y-axis over time, which the authors attribute to loosening of the anchoring mechanism since no clinical relevance could be sought.

## Introduction

The first generation mid-urethral slings (MUS) for female stress urinary incontinence (SUI) were introduced in 1996 with several modifications. The main purpose is to act as a “hammock” to support the middle part of the urethra [1,2] with the goal of achieving dynamic urethral kinking during increased abdominal pressure [3]. Currently, single-incision mini-sling (SIS) has gained popularity for its non-invasiveness. It has a shorter trajectory to avoid external needle passes through the groin or abdomen, with efficacy rates comparable to standard MUS at 1-year [4].

The MiniArc SIS system employs a self-fixing tip to provide immediate fixation into the obturator internus muscle. A 5-year long-term follow-up study done by Lo et al [5] shows satisfactory results, with an objective cure of 84.7% and subjective cure of 80%. The standard transobturator (TOT) approach, which was introduced by Delorme [6] to eliminate blind passage to the retroperitoneal space, was associated with neurological symptoms of groin pain, thigh numbness, and neurovascular injury. However, long-term outcome at 5-years using the Monarc TOT showed a satisfactory objective cure rate of 89.3% and subjective cure of 87.5% [7].

Ultrasonography has been able to demonstrate the anatomical location of the sling and the vesico-urethral junction in static and dynamic view [3,8–10]. It enables further understanding of the variable success rate of different sling systems through correlation of the mechanism and clinical outcomes [4]. In an ultrasonographic comparative study by Dietz et al [8] on tension-free vaginal tape (TVT) and TOT, the mode of action for continence seems identical for both- following the principle of dynamic urethral compression between the tape and symphysis pubis. The TOT has less effect on voiding function than TVT since TOT tapes form a bar rather than a loop underneath the urethra providing lesser lateral compression effect.

In contrast, an ultrasonographic comparative study by Lo et al [4] on MiniArc SIS and Monarc TOT showed dynamic urethral kinking and urethral compression as attributes to the continence effects. The urethra in the MiniArc group was significantly impinged with longer urethral core diameter in comparison to Monarc. The maximum urethral closure pressure (MUCP) was observed to be higher as well. Yet, objective ad subjective cure rates were comparable. Thus, the present study aims to present a follow-up study at 3-years time to determine the ultrasonographic changes that occurred. We hypothesized the MiniArc tape to have changed its position from the original fixation point despite having a self-fixing tip design since 5-year results showed declining cure rates.

## Materials and methods

This is a follow up study of the previous prospective study [4] on patients with symptomatic SUI and urodynamically confirmed urodynamic stress incontinence (USI) from March 2010 to December 2011. Approval from the institutional review board of Chang Gung Memorial Hospital was obtained (No. 201700320B0). Patients with neurological bladder dysfunction, pelvic organ prolapse >stage II based on the International Continence Society (ICS) grading system, and post-void residual (PVR) urine of >100 mL were excluded.

The type of MUS used depended on the patient’s informed choice. Counseling was done regarding the surgical procedure, cost, potential risk, benefits, and complications after surgery. A written informed consent was procured from all patients in the study. Preoperative evaluation as described previously [4] included detailed medical history, pelvic examination, 1-hour pad test, cough stress test (CST), urine analysis, and multichannel urodynamic studies. Patients were asked to complete a 72-h voiding diary and answer validated questionnaires such as UDI-6 [11], Incontinence Impact Questionnaire (IIQ-7) [12] and Pelvic Organ Prolapse/ Urinary Incontinence Sexual Questionnaire (PISQ-12) [13,14]. Multichannel urodynamic study followed the standardized protocol set by the ICS, which includes filling and voiding cystometry with surface electrode electromyography, urethral pressure profile and uroflowmetry [15].

USI was defined as involuntary leakage of urine during increased intra-abdominal pressure in the absence of detrusor contraction during filling cystometry. Abnormal PVR was defined as PVR of >150 mL or >20% of the post-void volume [16]. Detrusor overactivity (DO) was defined as spontaneous or provoked involuntary detrusor contraction during filling cystometry producing a waveform pattern of variable duration and amplitude on cystometrogram.

The MiniArc^TM^ (AMS, Minnetonka, MN, USA) was used to represent the SIS group. The surgical procedure was performed as described by Kennelly [17] with some modifications. An absorbable 1–0 polyglactin suture was placed near the right anchor of the sling. On sling placement, the sling was placed flat against the mid-urethra with no space in-between. Then, approximately 2 cm of the suture was left protruding from the vaginal wall. The purpose of the suture was to relieve consequences of voiding dysfunction from over-tensioned slings in the immediate post-operative period without compromising continence effects that can be done in an outpatient setting [18]. The traditional TOT group used the Monarc^TM^ (AMS, Minnetonka, MN, USA). Incision, dissection, tunneling, and delivery of suburethral sling were performed following the procedural guidelines of Davila [19]. Tightening of the sling was achieved by ensuring sufficient space to place a Metzembaum scissors between the urethra and the sling. All procedures were done by the senior author, who had performed 120 cases of Monarc and 20 cases of MiniArc prior to initiation of the study.

Cystoscopy was performed on all patients at the end of procedure to ensure bladder integrity. Urine was drained after evaluation with no catheter indwelled. Post-operatively, PVR urine was checked with the use of a bladder scan. For PVR >20% the voided volume and/or >150 ml, introital ultrasound was performed to observe for urethral indentation or elevation, which indicated over-tensioned sling. If the sling was over-tensioned, a gentle downward pull on the TRS attached to the MiniArc was done with the use of a hemostatic clamp. Lengthening of the suture was a sign of sling motion [18]. While for Monarc, a urethral dilator was used to push the proximal urethra downwards if the sling was over-tensioned. Clean intermittent self-catheterization was taught to patients with PVR ≥150ml for 5 days prior to discharge. Follow up visits were at 1 week, 1 month, 3 months, 6 months, and annually thereafter. PVR urine measurements, urinalysis, pelvic examination, and physiotherapy such as pelvic floor muscle training (PFMT; i.e. Kegel exercise) with or without biofeedback were performed at every visit. Multichannel urodynamic study, introital ultrasound, and validated questionnaires (UDI-6, IIQ-7 and PISQ-12) were done on 1^st^ and 3^rd^ year follow-up. Patients who were unable to follow-up were called through telephone by an accredited nurse to establish status of well-being.

The 3D introital ultrasound evaluated mobility of the sling (T) and bladder neck (BN), sling tightness, and percentile of the sling in relation to the urethra and the presence of urethral kinking using Philips ultrasound system (Philips HD11XE; Philips Ltd., Netherlands) [4]. This was performed at 6^th^ months and 3^rd^ years after surgery. The patient was placed in a semi-supine-lithotomy position with the transducer placed between the labia majora and below the external urethral orifice.

**Fig 1:**
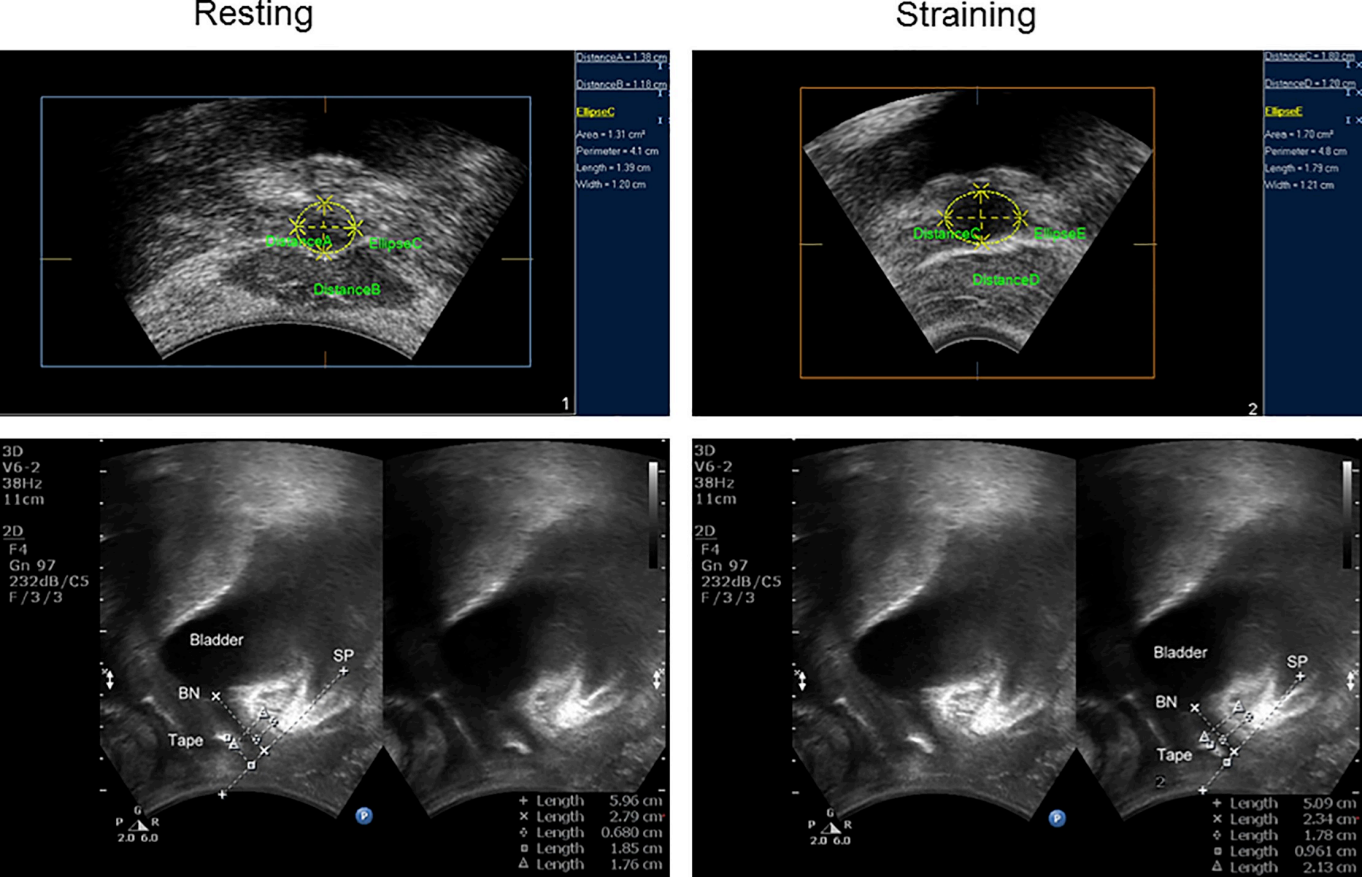
3D and 2D introital ultrasound imaging revealing the lower urinary tract and mid-urethral sling during rest and straining; BN, bladder neck; SP, Symphysis Pubis.

**Fig 2:**
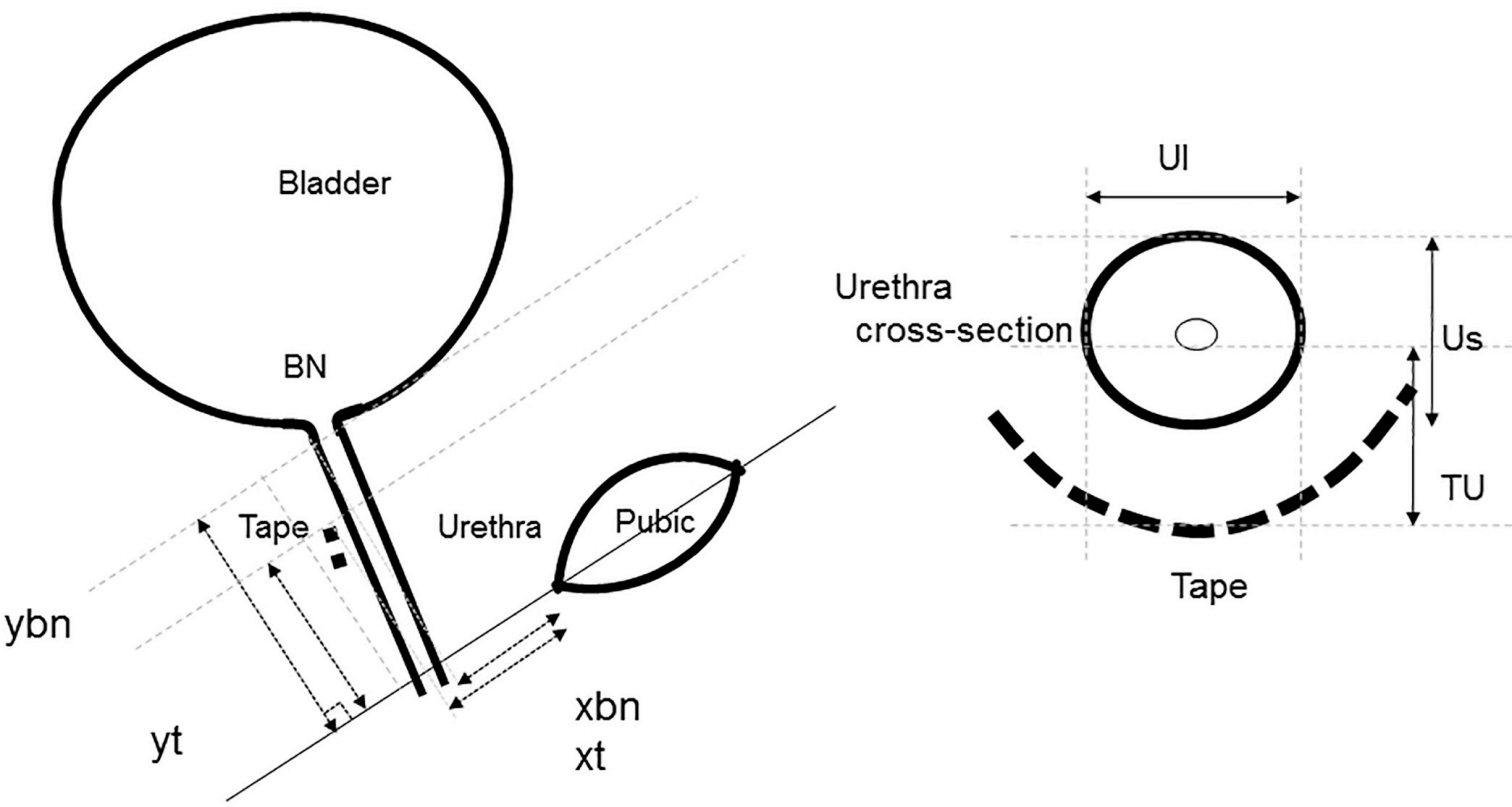
Graphical representation of the ultrasound parameters exploring the sling (xt, yt), BN position (xbn, ybn) and tape tension (TU, Ul, Us); BN Bladder Neck, distances between the mid position of the sling and center of urethral core (TU), the shortest (Us) and, longest (Ul) diameters, (xt, yt) x and y axis exploring the sling and (xbn, ybn) x and y axis exploring the bladder neck position.

The ultrasound parameters being measured were similar to our previous study [4,20]. The parameters for the sling (xt, yt) and BN position (xbn, ybn) (Figs 1 and 2) were measured in a sagittal plane using the rectangular coordinate system. The lower margin of the symphysis pubis at rest and maximum Valsalva serve as the reference marker [4,20], and the inferior margin of the sling as the reference point. The “xt” refers to the x-axis of the sling, “yt” as the y-axis of the sling, “xbn” as the x-axis of the bladder neck and “ybn” as the y-axis. Valsalva measurements were done 3 times with the most effective recording utilized. To compute for mobility of the sling (MobilityT) and bladder neck (MobilityBN), the formula √[(xtval—xt_rest_)^2^ + (ytval−yt_rest_)^2^] and √[(xbnval—xbn_rest_)^2^ + (ybnval−ybn_rest_)^2^] was used respectively. The formula was derived from the Pythagorean theorem, which states that the square of the hypotenuse (the side opposite the right angle) is equal to the sum of the squares of the other two sides. The acronym “val” represents the value during Valsalva maneuver and “rest” represents the value at rest [21].

Tightness of the sling was assessed in the axial plane of the urethra at rest. The major (Ul, longest diameter of urethra core at cross section) and minor axis (Us, shortest diameter of urethra core at cross section) of the urethra, and the distance between the position of the sling and center of urethral core (TU) were measured during rest and maximum Valsalva (Figs 1 and 2). The 3D rendering feature was used to ensure correct measurements of the urethral lumen in the axial plane. The presence of urethral kinking, seen as sling angularity or angle configuration of the urethra during maximum straining, provides imaging assessment on the continence effect of the sling [20]. The position of the sling was measured as a percentage in relation to the urethra. The distal end of the urethra is assigned to be 0% and the bladder neck as 100%.

Main outcome measure determined the change in position of the sling through measurement of the x- and y-axis at rest and during Valsalva maneuver. Secondary outcome assessed clinical end result measured as: Objective cure- absence of demonstrable leakage of urine on the cough stress test, provocative filling cystometry, and 1-hour pad test of a weight <2g; and Subjective cure- negative response to Urogenital Distress Inventory Six (UDI-6) (question 3) i.e. no urinary leakage on coughing, laughing or sneezing [9].

Paired *t*-test and Chi-square test or Fisher’s exact test were used for continuous variables and categorical variables respectively with p<0.05 as statistically significant. The methods, definitions, units, and classification of complications conform to the standards recommended by the International Urogynecological Association (IUGA) and the International Continence Society (ICS) [22].

## Results

**Fig 3:**
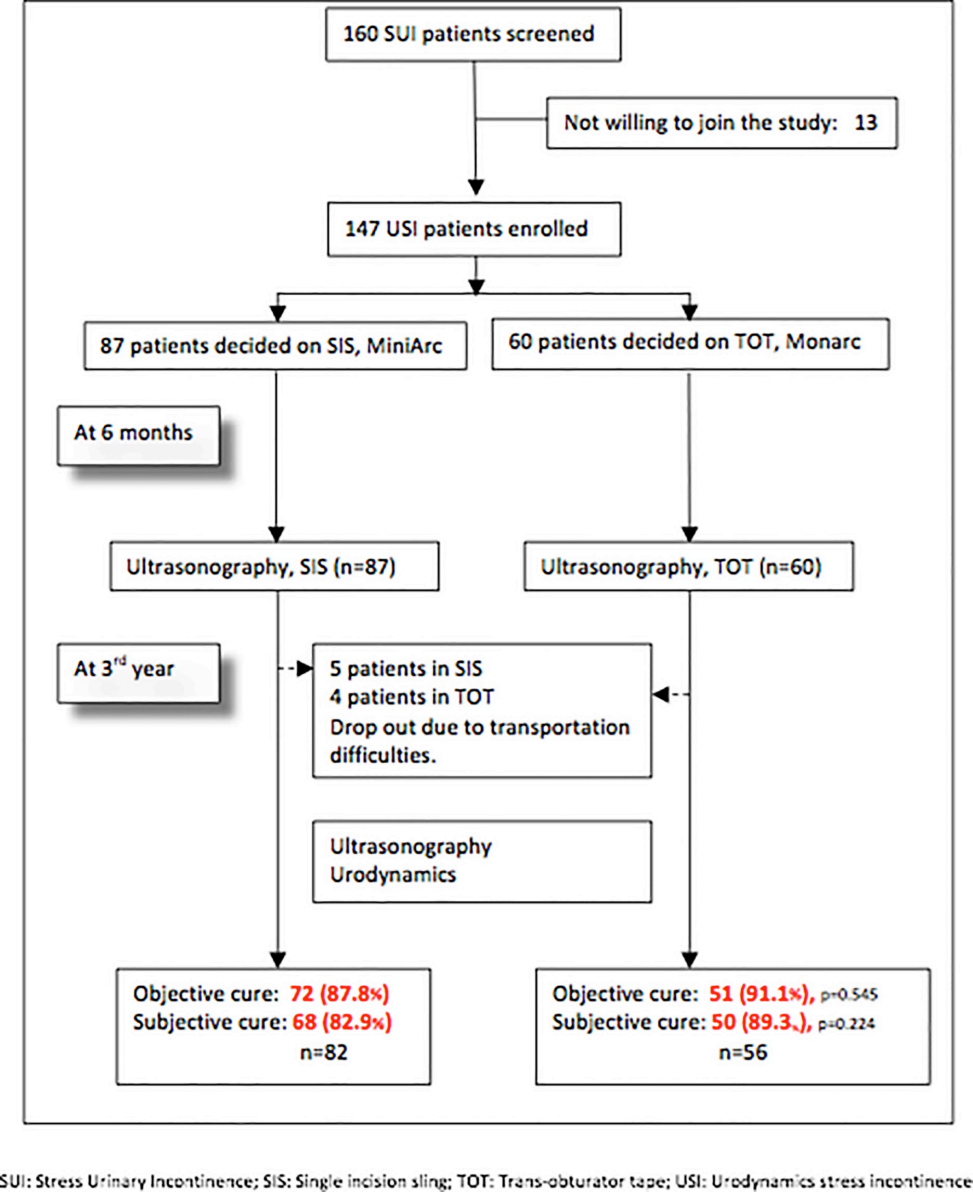
Flow chart of patients.

There were 147 patients with USI included in the study. However, only 138 patients were evaluated after 3 years, since 9 patients did not go on the scheduled follow-up due to transportation difficulties (Fig 3). Telephone interview was made to ensure safety of the 9 patients. The 138 patients were divided into MiniArc and Monarc group, depending on their choice of surgery. Objective cure for MiniArc was 88% (72/82) and Monarc, 91% (51/56). Subjective cure for MiniArc was 83% (68/82) and Monarc, 89% (50/56) (Fig 3). In addition, 6 patients in the MiniArc group had TRS manipulation immediately after surgery.

Patient demographics for each group are detailed in Table 1. The patient’s age, body mass index (BMI), parity, menopausal status, previous surgeries, and complication rates were comparable between the 2 groups. BMI was within normal limit at 24–25 kg/m^2^ for both groups. There were no complications involving bladder perforation and mesh extrusion/exposure noted throughout the follow-up period. However, 1 patient had intra-operative bladder perforation in the Monarc group.

**Table 1:** Patient demographics.

	Miniarc, n = 87	Monarc, n = 60	P value
Age (years)^a^	58.2±10.9	55.9±11.1	.181
BMI (kg/m^2^)^a^	24.2±3.4	25.1±3.7	.461
Menopausal (n)	58 (68.2%)	37 (65.4%)	.533*
Parity (n)^a^	3.0±1.3	2.9±1.1	.411
Previous surgery	13	8	.784*
VTH + Prolift total +A-P	3	3	
VTH + Perigee + SS + A-P	5	5	
Laparoscopic Burch	1	0	
Mid-urethral sling	3	0	
Needle suspension	1	0	
Operating time (mins) ^a^	32.5±11.2	35.3±22.5	.385
Intraoperative blood loss (mls) ^a^	26.9±26.6	28.7±19.2	.513
Median follow up period (month)	62.8±7.8 (60.7–64.8)	65.9±6.9 (64.4–76.7)	
Complication			
Bladder perforation	0	1	.408**
Sling extrusion/exposure (n)	0	0	-
Voiding dysfunction	0	0	
Post-operation hospital stay (days) ^a^	1.22±0.71	1.29±0.62	.479
Objective cure, 3^rd^ year (%)	72/82 (87.8%)	51/56 (91.1%)	.545*
Subjective cure, 3^rd^ year (%)	68/82 (82.9%)	50/56 (89.3%)	.224*

BMI, body mass index; VTH, vaginal total hysterectomy; SS, sacrospinous ligament fixation; A-P, anterior and posterior colporrhaphy.

^a^ Data listed as mean ± standard deviation or percentages in parentheses.

*Chi-square test

**Fisher exact test

The 3-year post-operative ultrasound evaluation is summarized in Table 2. Tape position measurements from 6 months to 3 years showed significant change in the position of MiniArc. MiniArc shifted its position in both x-axis [3.0 ±0.4 mm vs. 2.2 ±0.3 mm (p = 0.02) at rest; 2.6 ±0.3 mm vs. 1.6 ±0.3 mm (p<0.001) during valsalva] and y-axis [3.5 ±0.5 mm vs. 2.0 ±0.3 mm (p<0.001) at rest; 3.3 ±0.5 mm vs. 2.9 ±0.3 mm (p = 0.037) during Valsalva]. The position of the bladder neck showed minimal change over 3 years in both groups, but statistical analysis showed no significant difference. Comparison of urethral measurements on Ul and Us at rest and during Valsalva between 2 groups showed comparable results. The phenomenon of urethral kinking, as had been previously described, was demonstrated in 50 (61%) patients belonging to the MiniArc and 33 (59%) in Monarc group. A sample video clip showing urethral kinking of MiniArc is depicted in “S1 File Video”. The position of the sling in relation to the urethra was 60% from the distal end for both MiniArc and Monarc.

**Table 2:** The three-year ultrasound findings of anti-incontinence procedures (resting and maximum valsalva).

			Rest			Valsalva			*Resting vs straining*	
			Miniarc n = 82	Monarc n = 56	P*	Miniarc n = 82	Monarc n = 56	P*	P**	P**
Tape position	6^th^ month	xt (mm)	17.5 ±3.1	16.8 ±2.8	.241	21.9 ±3.2	22.1 ±3.4	.613	**<0.001**	**<0.001**
		yt (mm)	16.8 ±3.2	17.0 ±2.8	.511	5.7 ±3.1	5.4 ±2.8	.538	**<0.001**	**<0.001**
		MobilityT	-	-	-	11.9 ±1.0	12.5 ±1.1	.227	-	-
	3 year	xt (mm)	20.5 ±3.0	19.0 ±2.9	.077	24.5 ±3.4	23.7 ±3.2	.141	**<0.001**	**<0.001**
		yt (mm)	20.3 ±3.1	19.6 ±3.3	.126	9.0 ±2.2	8.3 ±2.2	**.027**	**<0.001**	**<0.001**
		MobilityT				13.0 ±1.2	12.2 ±0.9	**.011**		
	position	δxt (mm)	3.0 ±0.4	2.2 ±0.3	**.0.02**	2.6 ±0.3	1.6 ±0.3	**<0.001**	.426	.331
	shifted	δyt (mm)	3.5 ±0.5	2.0 ±0.3	**<0.001**	3.3 ±0.5	2.9 ±0.3	**.037**	.310	.651
	over 3^rd^ year	δMobilityT				1.1 ±0.4	0.3 ±0.3	**.001**		
	3^rd^ year	Percentile of urethral sling location (%)	59.3± 10.1	60.8± 12.8	.401	-	-	-	-	-
	3^rd^ year	Urethral kinking (%)^a^	-	-	-	50 (60.9%)	33 (58.9%)	.860	-	-
Bladder neck position	6^th^ month	xbn (mm)	5.7 ±2.8	5.8 ±2.1	.514	19.5 ±3.1	20.2 ±3.5	.417	**<0.001**	<0.001
		ybn (mm)	29.4 ±2.7	29.2 ±3.3	.641	21.5 ±3.7	21.7 ±3.9	.641	**<0.001**	<0.001
		MobilityBN	-	-	-	15.9 ±1.5	16.2 ±1.9	.181	**-**	-
	3 year	xbn (mm)	6.3 ±2.1	6.4 ±2.2	.331	20.3 ±3.3	20.9 ±3.1	.331	**<0.001**	<0.001
		ybn (mm)	30.2 ±3.8	29.9 ±3.5	.227	22.5 ±4.1	22.6 ±3.4	.670	**<0.001**	<0.001
		MobilityBN				16.0 ±1.5	16.0 ±1.6	.882		
	position	δxbn (mm)	0.6 ±0.2	0.6 ±0.2	.775	0.8 ±0.3	0.7 ±0.2	.451	.178	.461
	shifted	δybn (mm)	0.8 ±0.3	0.7 ±0.2	.310	1.0 ±0.3	0.9 ±0.2	.470	.514	.347
	over 3^rd^ year	δMobilityBN				1.2 ±0.2	1.1 ±0.2	.207		
Urethra cross- section	3^rd^ year	TU (mm)	5.2 ±2.1	5.4 ±2.1	.374	3.8 ±1.3	3.9 ±1.4	.751	**<0.001**	<0.001
		δTU				1.4 ±0.5	1.5 ±0.7	.331		
	3^rd^ year	Ul (mm)	15.9 ±2.7	15.6 ±2.9	.713	20.1 ±2.3	19.9 ±2.7	.531	**<0.001**	<0.001
		δUl				4.2 ±0.6	4.3 ±0.5	.513	**-**	-
		Us (mm)	14.2 ±2.5	14.3 ±2.3	.618	10.6 ±1.4	10.8 ±1.7	.401	**<0.001**	<0.001
		δUs				3.4 ±0.5	3.5 ±0.5	.211		

xt = distance between sling and axis perpendicular to central line of symphysis (cephalocaudal position); yt = distance between sling and central line of symphysis (ventrodorsal position); MobilityT = √[(xtval—xt_rest_)^2^ + (ytval−yt_rest_)^2^]; δxt = xt(3^rd^ year)–xt (6^th^ months); δyt = yt(3^rd^ year)–yt (6^th^ months); δMobilityT = MobilityT (3^rd^ year)–MobilityT (6^th^ months); xbn = distance between bladder neck and axis perpendicular to central line of symphysis (cephalocaudal position); ybn = distance between bladder neck and central line of symphysis (ventrodorsal position); MobilityBN = √[(xbnval—xbn_rest_)^2^ + (ybnval−ybn_rest_)^2^]; δxbn = xbn(3^rd^ year)–xbn (6^th^ months); δybn = ybn(3^rd^ year)–ybn (6^th^ months); δMobilityBN = MobilityBN(3^rd^ year)–MobilityBN (6^th^ months);TU = distance between the sling and center of urethral core; δTU = (TU_rest_−TU_val_);; Ul = longest diameter; δUl = (Ul_val_−Ul_rest_); Us = shortest diameter; δUs = (Us_val_−Us_rest_)

^a^ sling angularity noted on ultrasonography in patients during maximum straining

Data listed as mean±standard deviation

val represents the value during the Valsalva maneuver and rest represents the value at rest.

*p value for between-group comparison

** p value for within-group comparison.

Pre- and post- urodynamic findings were shown in Table 3. At 3 years post-surgery, both groups have comparable urodynamic parameters. Values for Qmax, RU, CC, FUL, and Dmax were not statistically different amongst the 2 groups and were within the normal range. MUCP increased post-operatively for both groups but did not statistically differ when compared against each other. USI significantly improved for both groups (p<0.001), however, recurrence was observed in 10 patients (12%) for MiniArc and 5 (9%) for Monarc. Two patients in the MiniArc and two in the Monarc groups had de novo detrusor overactivity (DO). Abnormal PVR was not observed in any patient in either group from 6 months post-operative up to the present time at 3 years. No patient had bladder outlet obstruction or detrusor underactivity. Validated subjective questionnaires (UDI-6, IIQ-7 and PISQ-12) showed significant improvement in quality of life for both procedures at 3 years (Table 3).

**Table 3:** Pre and post surgery urodynamics UDI-6, IIQ-7, PISQ-12 comparison between Miniarc and Monarc.

	Pre-OP			Post-OP, 3^rd^ year			*Within group*	
	Miniarc n = 82	Monarc n = 56	P Between group	Miniarc n = 82	Monarc n = 56	P Between group	P	P
Qmax	27.8±10.3	27.3±11.1	.362	26.1±10.4	25.9±10.8	.717	.218	.201
RU	33.4±28.5	37.4±37.6	.216	35.2±26.7	39.2±27.1	.368	.511	.472
CC	410.7±118.2	408.1±101.4	.316	402.5±98.1	404.3±108.4	.227	.216	.716
MUCP	72.2±22.9	69.4±27.4	.537	76.6±31.2	72.9±27.3	.181	.176	.312
FUL	22.2±5.1	23.4±6.2	.419	22.9±5.2	23.7±5.1	.482	.519	.661
Dmax	14.5±11.4	14.7±12.9	.631	15.9±9.8	15.7±13.0	.611	.216	.336
USI	82 (100%)	56 (100%)		10 (12.2%)	5 (8.9%)	.545	<0.001	<0.001
DO	0	0		2 (2.4%)	2 (3.6%)	.536	.316	.315
BOO	0	0		0	0			
DU	0	0		0	0			
UDI-6	11.8±3.8	11.2±3.7	.597	4.7±2.9	4.6±3.1	.575	<0.001	<0.001
IIQ-7	14.9±4.1	13.2±4.1	.153	4.3±2.7	4.0±2.8	.388	<0.001	<0.001
PISQ-12	24.0±4.7	23.9±5.1	.431	27.6±4.3	27.9±4.2	.661	.002	<0.001
	n = 34	n = 24		n = 34	n = 24			

Qmax, maximum urinary flow (m/s); RU, postvoid residual urine (ml); CC, cystometric capacity (ml); MUCP, maximum urethral closure pressure (cmH2O); FUL, functional urethral length (cm); Dmax, detrusor pressure at maximum flow (cmH2O); USI, urodynamic stress incontinence; DO, detrusor overactivity; BOO, bladder outflow obstruction; DU, Detrusor underactivity; UDI-6, Urinary Distress Inventory; IIQ-7, Incontinence Impact Questionnaire; PISQ-12, Pelvic Organ Prolapse/ Urinary Incontinence Sexual Questionnaire

Data listed as mean ± standard deviation or percentages in parentheses.

*Chi-square test

**Fisher exact test

## Discussion

This follow-up study evaluated the mid-term outcome of two forms of anti-incontinence procedures. The ultrasound morphology and its clinical correlations were studied among women who had undergone MiniArc^TM^ or Monarc^TM^ surgery as treatment of SUI.

The curative effect of both mid-urethral slings are likely due to mechanical compression of the urethra by the sling, and/or kinking of the urethra around the sling [20]. During increased intra-abdominal pressure, both the tape & urethra are pushed away from the symphysis pubis with the sling serving as a back-board for compressive effect. In a sonographic study on transobturator slings, it was noted that a narrow gap between the tape and symphysis pubis is advantageous for the cure of stress and urge incontinence, and that overcorrection is highly unlikely due to the direction of force vectors resulting from the course of the tape passing through the obturator foramen [10]. The less compressive effect of TOT has been demonstrated in the study by Dietz et al when compared with TVT [8] and similarly, in the previous study by Lo et al when compared with MiniArc at 1-year [4].

To demonstrate sling tightness, Dietz et al [10] describes that a narrower gap between the sling and symphysis pubis and a more acute sling angle suggest greater urethral compression and may indicate sling “tightness”. In addition, Lo et al [4] demonstrates that a longer resting Ul and shorter resting Us on the urethra suggests an impinged/compressed urethra, and indicate sling “tightness” as well. The present study has shown both MiniArc and Monarc having comparable urethral measurements and urethral kinking in approximately 60% of patients. The previously observed impinged urethra with MiniArc at 1-year [4] was no longer evident at present. The position change of MiniArc noted in both x- and y-axis over time could have been due to its anchoring system since the mesh used was the same. Other causes such as tissue mobility and mesh stretching can be considered as well. In vitro testing of porcine models on the different anchoring system of mini-slings has shown the MiniArc SIS to have the lowest pull-out force, making it prone to dislocation [23]. It would be ideal to actually demonstrate and prove that the loosening of the tape was related to the anchoring tip, but is beyond the scope of the study. What the study can offer are the different ultrasound measurements that indicate sling position, movement, and tension.

Bladder neck mobility is likely a prerequisite for dynamic compression of the urethra. For slings to work best, a mild degree of hypermobility between 2.5 and 4cm is needed, however this concept by Dietz has no proof. Even minimal mobility can be curative in patients [9]. The bladder neck mobility of the study is 16mm for both MiniArc and Monarc yielding satisfactory objective and subjective cure rates at 88% and 83% respectively.

The location of the sling in relation to the urethra has been associated with recurrence of SUI on transobturator tapes placed proximal to the urethra [24] Yet, Chantarasorn et al [10] reports that the location of the sling in relation to the urethra does not affect surgical outcomes. The study exhibited that the position of both slings in relation to the urethra did not significantly differ, instead surgical outcomes depend more on the fixation point of the sling.

Studies have suggested that excess body weight can increase abdominal and bladder pressure, and affect the mobility of the urethra. [25]. There were a few patients in the study were overweight, but the study calculated for the mean BMI for each group. Whether BMI affected outcomes of MUS was not the focus of the study.

De novo detrusor overactivity was observed in 2.4% of patients in MiniArc and 3.6% of patients in Monarc. It probably occurred secondary to age-related effects on vaginal mucosa shrinkage and atrophy of urethral tissue or due to infection and other diseases unrelated to the sling surgery. A 10-year follow up study on patients after mid-urethral sling surgery had 3.6% rate of de novo urgency [26].

Since MiniArc has been removed from the market, other single incision slings such as the Solyx (Boston Scientific, USA) and Ophira (Promedon, Argentina) that utilize the same principle of self-fixing design anchoring tips. There have been no mid-term outcomes reported on these new slings; therefore, the data from this current study can serve as a reference for these SIS systems. Long-term assessment of these various slings is recommended in order to design an anchoring mechanism that will remain fix in place over time.

The weakness of this study is that it is a non-randomized cohort group. The senior author performed the ultrasonographic measurements and as such, human error and bias could not be eliminated as well as measurement errors. Strengths include being a pragmatic clinical trial, which evaluated the effectiveness of the intervention in real-life clinical practice; mid-term study, and ultrasonographic imaging that allowed observation of the position and movement of the sling.

## Conclusion

Maintenance of continence rates of mid-urethral slings depends on the compressive effect of the sling on the urethra, urethral kinking, and sling fixation. From 6months to 3 years, MiniArc changed its position in both x- and y-axis over time, which the authors attribute to loosening of the anchoring mechanism since no clinical relevance could be sought.

## Supporting information

S1 File Video Dynamic urethral kinking phenomena at Valsalva after a MiniArc surgery. (M4V)
